# Practical application of synthetic head models in real ballistic cases

**DOI:** 10.1007/s00414-021-02671-3

**Published:** 2021-08-15

**Authors:** F. Riva, T. Fracasso, A. Guerra, P. Genet

**Affiliations:** 1 Centre Universitaire Romand de Médecine Légale Lausanne-Geneva, University Hospital of Lausanne, Lausanne, Switzerland; 2 Ecole Des Sciences Criminelles, University of Lausanne, Lausanne, Switzerland; 3 Centre Universitaire Romand de Médecine Légale Lausanne-Geneva, University Hospital of Geneva, Geneva, Switzerland

**Keywords:** Forensic, Wound ballistic, Simulant, Synthetic model, Headshot

## Abstract

In shooting crimes, ballistics tests are often recommended in order to reproduce the wound characteristics of the involved persons. For this purpose, several “simulants” can be used. However, despite the efforts in the research of “surrogates” in the field of forensic ballistic, the development of synthetic models needs still to be improved through a validation process based on specific real caseworks. This study has been triggered by the findings observed during the autopsy performed on two victims killed in the same shooting incident, with similar wounding characteristics; namely two retained head shots with ricochet against the interior wall of the skull; both projectiles have been recovered during the autopsies after migration in the brain parenchyma. The thickness of the different tissues and structures along the bullets trajectories as well as the incident angles between the bullets paths and the skull walls have been measured and reproduced during the assemblage of the synthetic head models. Two different types of models (“open shape” and “spherical”) have been assembled using leather, polyurethane and gelatine to simulate respectively skin, bone and soft tissues. Six shots have been performed in total. The results of the models have been compared to the findings of post-mortem computed tomography (PMCT) and the autopsy findings.Out of the six shots, two perforated the models and four were retained. When the projectile was retained, the use of both models allowed reproducing the wounds characteristics observed on both victims in terms of penetration and ricochet behaviour. However, the projectiles recovered from the models showed less deformation than the bullets collected during the autopsies. The “open shape” model allowed a better controlling on the shooting parameters than the “spherical” model. Finally, the difference in bullet deformation could be caused by the choice of the bone simulant, which might under-represent either the strength or the density of the human bone. In our opinion, it would be worth to develop a new, more representative material for ballistic which simulates the human bone.

## Introduction

### Wound ballistics in forensic medicine

In crimes involving shooting incidents, the traces collected on the scene, those recorded during the examination of the victim (external examination and/or the autopsy of the corpse) and those analysed in the laboratory should be combined in order to allow a complete shooting scene reconstruction. This includes positioning of the traces and relevant ballistic evidence, like spend cartridge cases or bullets, trajectory estimation and representation, gunshot residues (GSR) samples as well as wound ballistic findings. However, the integration of wound ballistics findings into the scene needs a correct interpretation of gunshot wounds characteristics [1–4]. Because of the big variety of the existing ammunitions and firearms, the projectiles have different properties (energy, velocity, form, composition, etc.) and behave differently in the human body [2] creating different types of wounds [5–7]. The variability in terms of wounding is not only related to the penetration depth or the transfer of energy, but depends also on the deviation in soft tissues [8–11] as well as on the deviation caused by the interaction with bones [12]. These aspects are particularly important when a shooting trajectory estimation involving a victim is integrated into a crime scene model [8]. In order to provide a correct wound interpretation, ballistic tests are therefore often performed in conditions as close as possible to the questioned case [13].

### Shooting tests in wound ballistics

During the reconstruction of shooting crimes, multiple hypotheses are often emitted, especially when several shots have been fired or when the version of the suspect must be proven. For example, it is important to know if the observed wound on a victim has been caused by a direct shot or a ricocheted or deviated projectile. These alternatives are evidently considered in a different way from a legal point of view. Shooting tests are thus often performed with the intention to reproduce the wound characteristics of the injuries. They permit frequently to confirm or to exclude the hypothesis emitted at the beginning of such shooting investigations.

The set-up of a wound ballistics test is usually driven by the background information, namely the wound characteristics described by the forensic pathologist and the crime scene findings [1]. On the basis of the available information, the shooting tests target to reproduce as faithfully as possible the wound characteristics taking into account the types of organs and biological structures hit by the projectile. To counter the ethical and legal complications presented when using human cadaveric tissue [14], several “surrogates” or “simulants” are often used. The mostly used are surrogates concerning soft tissues (e.g. skin or muscle tissue) and bones. More recent publications also targeted the lung parenchyma, which provides different resistance characteristics compared to muscle tissue for example [15].

A variety of surrogates have already been used to model ballistic trauma; these include corpses, animals and synthetic or non-humane biological materials [5, 16, 17]. Corpses or animals show several constraints, namely the ethical issue related to their use, the availability of specimens and the reproducibility of the results related to their heterogeneity [17, 18]. Contrariwise, synthetic or non-humane biological materials showed a satisfactory to good level of reproducibility [5, 18], in replacing human tissues like soft tissues (skin, muscle or brain tissue) or bones [1, 14, 19–33]. For ballistics tests, these synthetic or non-humane biological materials are usually used in a combined way by assembling them together in order to create a realistic wound model. Several models have already been subject of studies; most of them concerned the torso [16] or the head [1, 5, 23, 33, 34].

Despite the efforts of the last years in the research of “surrogates” in the field of forensic ballistic, the development of the above-mentioned models needs still to be improved through a validation process based on specific real caseworks comparisons.

### Aim of the study

The purpose of this paper is to further amend existing knowledge by comparing the performances of two synthetic head models described in the literature to the findings of ante- and post-mortem computed tomography (PMCT) and the autopsy findings of two victims of a well-documented shooting crime. Advantages, disadvantages and possible improvements of both models are compared and discussed.

## Materials and methods

This study has been triggered by the finding observed during the autopsy performed on two victims (two young males named here victim A and B) killed in the same shooting incident, with the same firearm, same ammunition, comparable shooting distance (small room) and with similar wounding characteristics. The questioned ammunition was a Geco calibre 6.35 mm Browning (0.25 Auto) equipped with a Full Metal Jacket (FMJ) projectile with a brass jacket. The 3.2 g (50 grains) bullet, showed a cannelure in the middle of its body. No precise information regarding the firearm was unfortunately available. Based on the general rifling characteristics of the bullet and the cartridge case, a limited list of potential candidate weapons was evocated. All the mentioned firearms were similar semi-automatic pistols, calibre 6.35 mm Browning (0.25 Auto) having a barrel length of approximately 5 cm (2″). The shooting took place in a confined indoor space with a distance of maximum a few meters between the shooter and its victims.

### Wounds characteristics and analysis of the ante- and post-mortem CT-scan

**Fig. 1:**
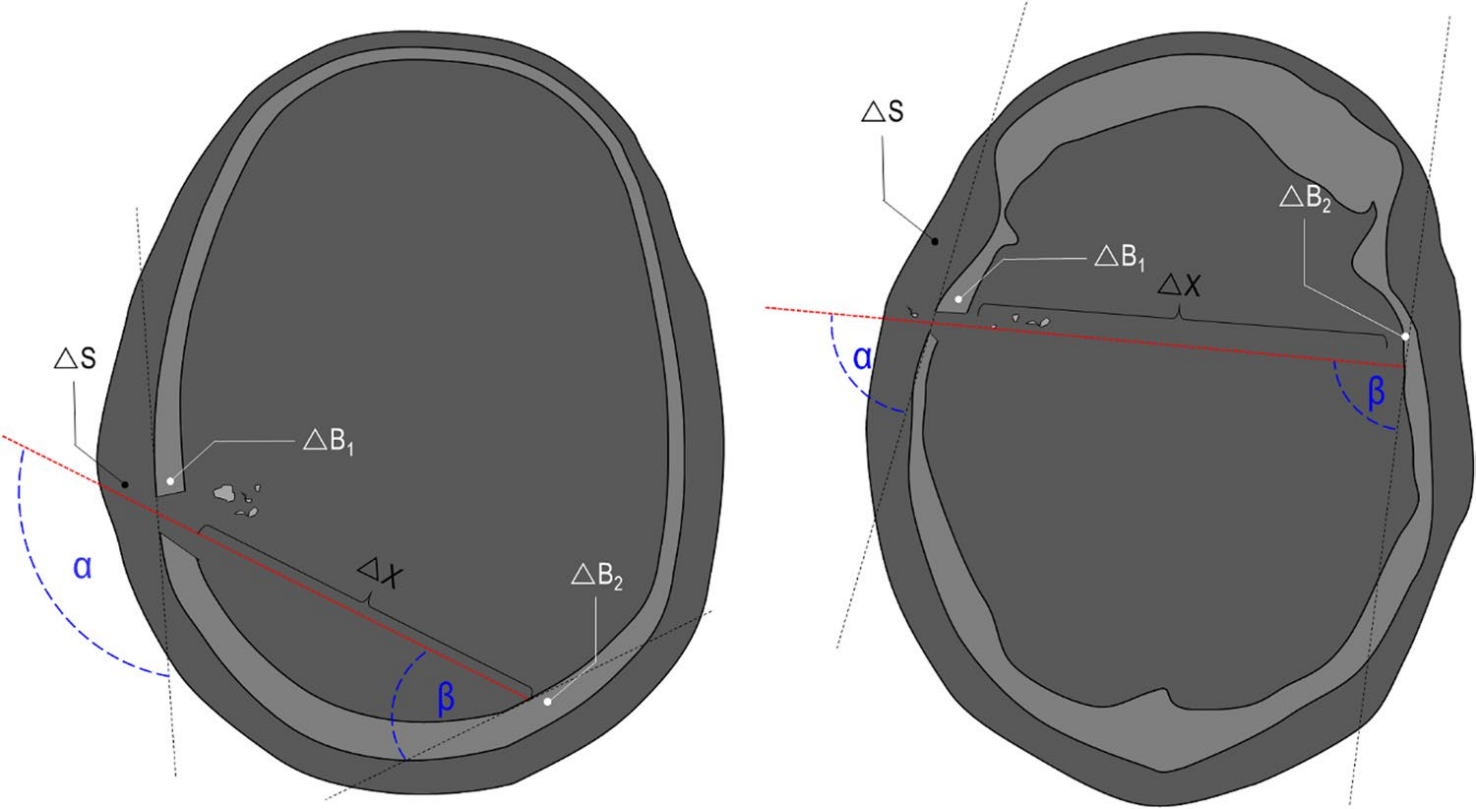
Schema of the wound tracks (red dotted line) of victim A (left) and victim B (right) visualised in the axial plane of the PMCT with illustration of the measured trajectory parameters: α, △S, △B_1_, △X, △B_2_, and β. α = incident shooting angle, △S = thickness of the skin and subcutaneous layers, ∆B_1_ = thickness of the temporal bone at the entry hole, △X = length of the projectile path in the brain before ricochet, △B_2_ = thickness of the occipital bone where the projectile ricocheted, β = incident angle. No particular metric proportion has been observed

Both victims have been shot into the head; the projectile penetrated the skull without perforating it. In both cases, the projectile was retained in the skull. The victims did not die on the crime scene and were transported to the regional hospital, where a computed tomography (CT) was performed and where they were recovered in intensive care. Victim A died after 1.5 days and victim B died after 2.5 days of hospitalisation. After their death, they were transported to our institute of legal medicine to perform a medico-legal autopsy. Before the external examination and the autopsy, a native post-mortem CT-scan (GE LightSpeed VCT64) of the corps was performed in supine position. For both victims (A and B), the wound track characteristics were summarized by combining the observations done on the PMCT and the autopsy findings. These observations have additionally been completed by the findings noticed at the hospital on the ante-mortem CT (Fig. 1).

**Fig. 2:**
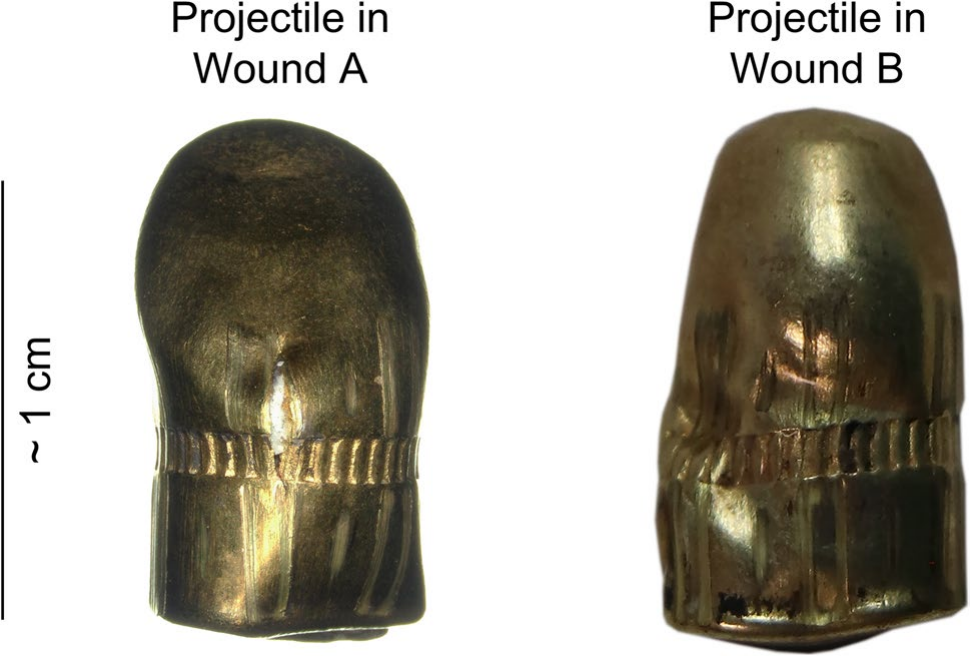
Projectiles recovered respectively in the Wound A (left) and the Wound B (right)

Victim A presented at the external examination at the right parieto-temporal region a round entry wound with a central skin defect, measuring 0.8 × 0.6 cm, associated to an abrasion ring and a contusion zone without any exit wound. At the ante-mortem and PMCT an irregularity of the skin at the same region was observed. This irregularity was associated to a loss of bone substance in form of a funnel-shaped open inwards at the right posterior parietal region measuring approx. 0.11 cm in the axial plane, associated to a few small bone fragments in the soft tissue of the scalp. The brain parenchyma showed an acute linear intra-parenchymal haemorrhage in the right parietal and temporal lobe extending to the left occipital lobe passing through the posterior horn of the right lateral ventricle, the right venous sinus and the middle line (approx. 12 cm of length). This linear haemorrhage extended further from the left occipital lobe to the left temporal lobe (approx. 8 cm of length). The presence of a hyper-dense foreign body, compatible with a projectile, was observed in both CT (ante-mortem and PM-CT); however, a discrepancy was noticed when comparing the position of this hyper-dense body between both scans. On the ante-mortem CT-scan, the projectile was situated in the left temporal lobe, while after death, during the execution of the PMCT, the bullet was situated in the left occipital region. The forensic pathologists concluded in a secondary migration of the projectile through the wound channel, which the projectile had created as passing through the brain parenchyma. The displacement of the projectile occurred probably along the gravity (Victim A was placed in a decubitus position as hospitalized; the brain parenchyma was weakened along the wound channel). This phenomenon, even if rarely observed, has already been documented in the past [35–40]. The direction of the trajectory of the projectile was therefore established as followed: from the right to the left, from the front to the back and from the top downwards with a ricochet in left occipital region redirecting the projectile towards the left temporal region. The projectile has been extracted during the autopsy, its weight was about 3 g and its surfaces showed a flat diagonal deformation starting from the top of the projectile and a less pronounced flat deformation on the opposite side of the projectile. The projectile was also slightly flattened at the base (Fig. 2 left).

Victim B presented at the external examination at the right temporal region, a round entry wound with a central skin defect, measuring 0.5 × 0.3 cm, associated to an abrasion ring and a contusion zone without any exit wound. At the ante-mortem and PMCT, an irregularity of the skin at the same region was observed. This irregularity was associated to a loss of bone substance in form of a funnel-shaped open inwards at the right temporal region measuring approx.1.2 cm in the axial plane, associated to a few small bone fragments in the soft tissue of the scalp. The brain parenchyma showed an acute linear intra-parenchymal haemorrhage in the right temporal lobe extending to the left temporal and parietal lobe passing through the middle line. The presence of a hyper-dense foreign body, compatible with a projectile, was observed in both CT (ante-mortem and PM-CT) in the left temporo-parietal region. Like in victim A, a little discrepancy was noticed when comparing the position of this hyper-dense body between both scans (ante- and post-mortem). In the ante-mortem CT-Scan the projectile was found 1 cm behind and slightly underneath the linear intraparenchymal haemorrhage. In the post-mortem CT-Scan the projectile was found 2 cm behind and slightly underneath the linear intraparenchymal haemorrhage; the projectile thus migrated between the CT-Scans. In both CT-Scans the projectile formed approximately a 40° angle with the haemorrhage. The direction of the trajectory of the projectile was therefore established as followed: from the right to the left, from the front to the back and from the bottom upwards, with a ricochet in left temporo-parietal region redirecting the projectile towards the inner part of the left temporo-parietal region of the brain. The detailed analysis of the projectiles at the PMCT was slightly limited by the metallic artefacts emitted by the projectiles. The projectile has been recovered during the autopsy (Fig. 2 right), its weight was about 3.1 g. The projectile showed several small deformations on its body and a main deformation at the base (flattened).

Both trajectories have been accurately described by the mean of the CT images. The thickness of the different tissues perforated or penetrated by the projectiles have been measured and are summarized in the Table 1. Figure 1 was added to illustrate the bullet paths through the head of victim A (left) and through the head of victim B (right).

**Table 1:** Thickness of the different tissues perforated or penetrated by the projectiles

	△S (cm)	∆B_1_ (cm)	△X (cm)	△B_2_ (cm)	α (°)	β (°)
Wound A	0.5–0.8	0.4–0.5	11.7^(1)^ + 8.2^(2)^	0.3	125	45
Wound B	0.8	0.3	12.8^(1)^ + 2.5^(2)^	0.4–0.6	79	73

^(1)^ Distance between the bone walls along the trajectory

^(2)^ Path length in the brain after the ricochet on the 2° bone wall

### Synthetic model set-up

To simulate the wound characteristics of the two cases described above, we choose to use two different synthetic models, both inspired from previous ballistic studies [1, 19, 20, 33, 41, 42], a so called “open shape” model and a so called “spherical” model.

#### The “open shape” model

**Fig. 3:**
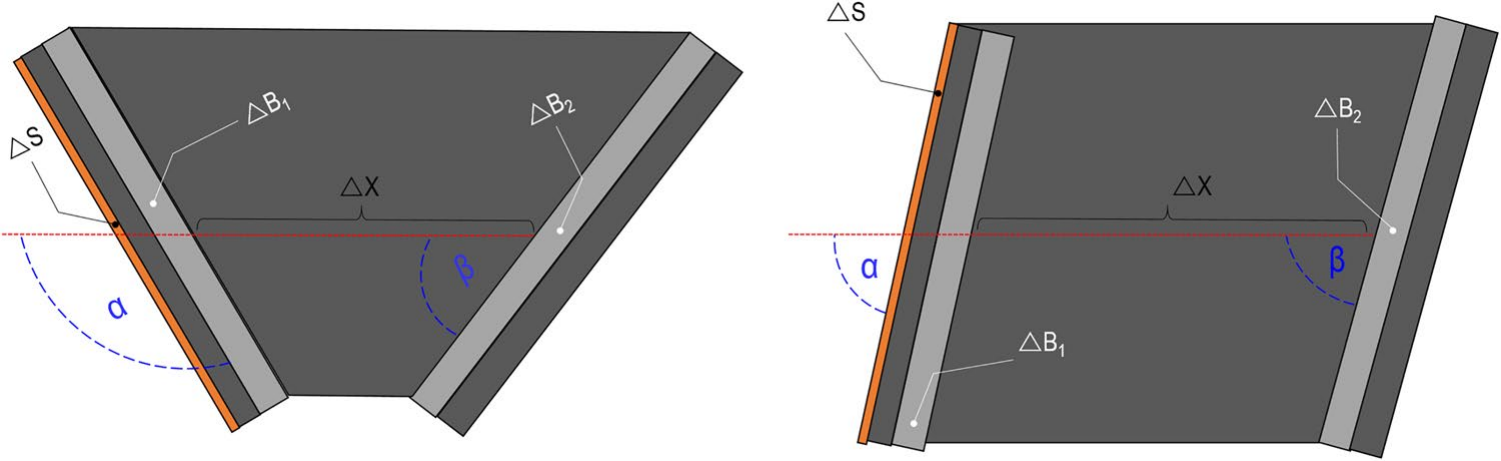
Graphical representation of the “open shape” model. Schemas of the models assembled for the simulation of wound tracks (red dotted line) of victims A (left) and victim B (right) visualised in the axial plane with illustration of the trajectory parameters: α, △S, △B_1_, △X, △B_2_, and β. α = incident shooting angle, △S = thickness of the skin and subcutaneous layers, ∆B_1_ = thickness of the first polyurethane plate, △X = length of the soft tissue portion along a straight trajectory before ricochet, △B_2_ = thickness of the second polyurethane plate, β = incident angle on the second polyurethane plate considering a straight trajectory. No particular metric proportion has been observed

The “open shape” model has been inspired by Riva et al. [1]; it is a multilayer model, where the layers are positioned sequentially in respect to the shooting direction (perpendicularly or with a precise incidence angle); no lateral walls to enclose the model are used. It is composed by (a) a layer of leather (cowhide, semi-finished chrome tanned upholstery “crust) with a thickness of 0.1 cm to simulate the skin in accordance with Jussilla et al. [27]; (b) a layer of ballistic gelatine 10% at 4 °C (Type 3, 250 Bloom number, Gelita, Eberbach, Germany) for the subcutaneous tissues, (c) a polyurethane plate with inorganic filler materials (Synbone AG, Malans, Switzerland) to simulate the skull wall (available in three different thickness: 5, 6 and 7 mm); (d) a portion of ballistic gelatine 10% at 4 °C as surrogate for the brain tissue and (e) a second polyurethane layer for the skull wall casted into the gelatine. The gelatine has been previously calibrated in according with the guidelines provided by Jussilla et al. [28]. The thickness of each single layer in the model corresponds to the thickness of each human tissue measured on the PMCT images considering the wound track position (Fig. 1 and Fig. 3). The angles between the trajectory followed by the projectile and the outer wall of the skull next to the entry wound (first impact at the skull), as well as next to the ricochet (second impact at the inner side of the skull) were called respectively angle α and β. They have likewise been measured on the PMCT images and have been reproduced on the models. All the mentioned dimensions are summarized in Table 2 according to the measurement illustrated in Fig. 1 and reproduced on the models represented in Fig. 3.

**Table 2:** Individual modelling of the questioned injuries A and B by the mean of the “open shape” models

	△S (cm)	△B_1_ (cm)	△X (cm)	△B_2_ (cm)	α (°)	β (°)
Wound track A	0.5–0.8	0.4–0.5	11.7^(1)^ + 8.2^(2)^	0.3	125	45
Model A	0.1^(3)^ + 0.5^(4)^	0.5	11.7 ^(5)^	0.5	125	45
Wound track B	0.8	0.3	12.8^(1)^ + 2.5^(2)^	0.4–0.6	79	73
Model B	0.1^(3)^ + 0.7^(4)^	0.5	12.8^(5)^	0.5	79	73

^(1)^ Distance between the bone walls along the trajectory

^(2)^ Path length in soft tissues after the ricochet on the bone wall

^(3)^ 0.1 cm cowhide, semi-finished chrome tanned upholstery “crust”[27]

^(4)^ Gelatine 10%

^(5)^ Distance between the polyurethane plates in the model along a straight trajectory

**Fig. 4:**
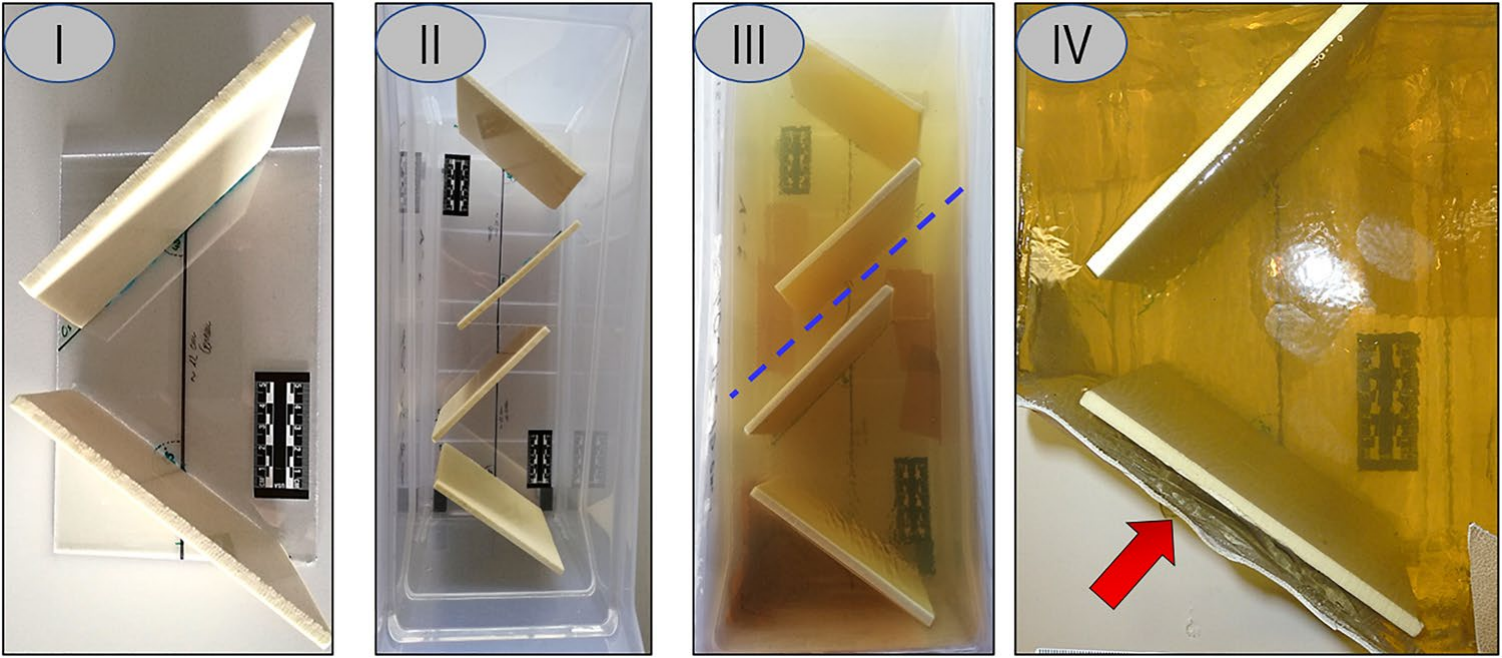
Individual model preparation. Part (**I**) illustrates the frame used to fix the polyurethane plates before the gelatine was added. Parts (**II**) and (**III**) represent both models in the mould respectively without and with gelatine. The dotted blue line represent the cut performed to separate the models from the single gelatine bloc. Part (**IV**) illustrates the finished model with the leather layer highlighted by the red arrow (direction of shooting from the bottom to the top of the image)

The models were prepared as followed: the polyurethane plates were placed according to the dimensions and characteristics listed in Table 1. The resulting blocs (Fig. 4 part I) were introduced into a mould (Fig. 4 part II) and the liquid gelatine (Type 3 of Gelita) prepared in according with Kneubuehl et al. [5] was poured into it (Fig. 4 part III). The models were placed for 48 h into a fridge at 4 °C. Once the models became solid, they were cut first in the middle in order to obtain two models with the same dimensions (Fig. 4 blue dotted line); then each model was cut close to the first polyurethane plate in order to obtain the suitable subcutaneous layer. The leather (0.1 cm) was finally added in the front part of the model (Fig. 4 red arrow) by heating the gelatine slightly (Fig. 4 part IV). At the end, the models were placed again into the fridge at 3–4° until the shooting tests.

Two “open shape” models with the same characteristics and dimensions have been assembled for each simulation (simulation of wound tracks of victims A and B); they were called A.1 and A.2 respectively for the simulation of the wound track of victim A and B.1 and B.2 for the wound track of victim B. The dimensions of the models were approximately 15 × 15 × 20 cm.

#### The “spherical” model

**Fig. 5:**
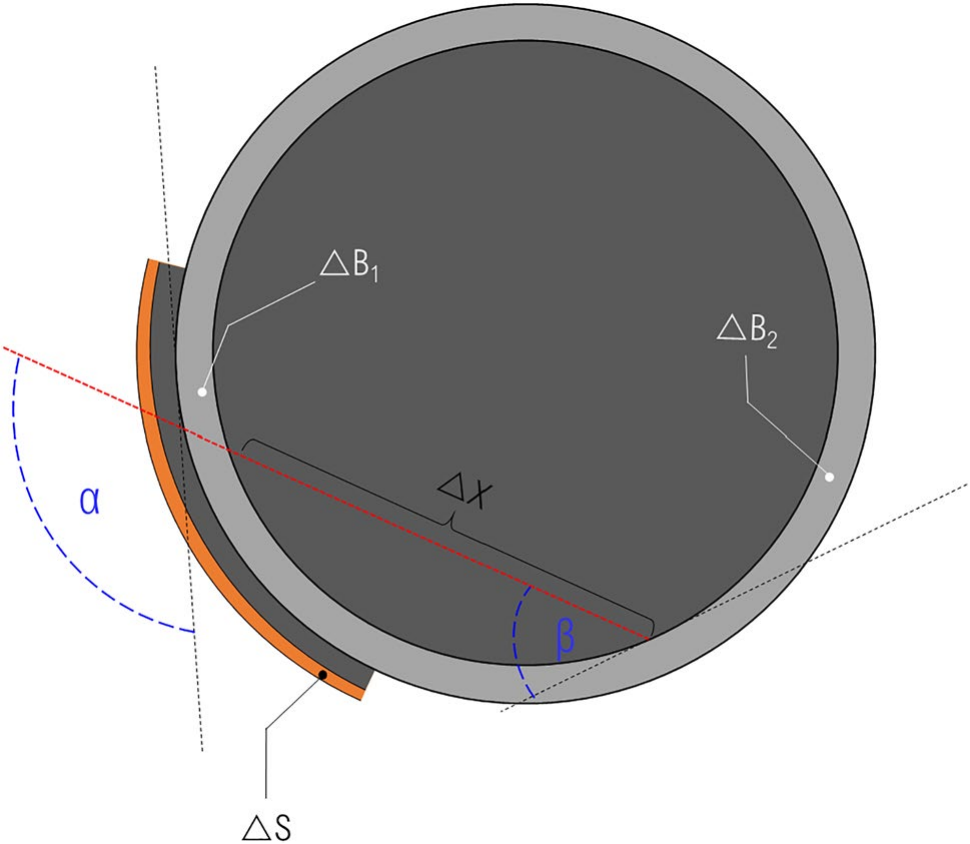
Graphical representation of the “spherical” model for the simulation of the wound track of the victim A. The red dotted line represents the projectile trajectory. The others trajectory parameters are: α, △S, △B_1_, △X, △B_2_, and β. α = incident shooting angle; △S = thickness of the skin and subcutaneous layers, △B_1_ = thickness of the first polyurethane plate, △X = length of the soft tissue portion along a straight trajectory before ricochet, △B_2_ = thickness of the second polyurethane plate; β = incident angle on the second polyurethane plate considering a straight trajectory. No particular metric proportion has been observed

The “spherical” model has already been used in several ballistic studies, especially in forensic science [5, 33, 43]. Similar head models have also already been used in wound ballistics tests [23, 25]. Its concept has been slightly adapted to the needs of our ballistic tests. The main body of the model is a 5 mm thick polyurethane sphere (Synbone AG, Malans, Switzerland) with a diameter of 19 cm fulfilled with calibrated ballistic gelatine 10% at 4 °C (Type 3, 250 Bloom number, Gelita, Eberbach, Germany). To simulate the skin and the subcutaneous tissues, a layer of 0.1 cm leather [27] has been attached on one layer of ballistic gelatine 10% at 4 °C; these layers have been placed on the polyurethane sphere as displayed in Fig. 5. Theirs values are summarized in Table 3.

**Table 3:** Individual modelling of the wound track A with the “spherical” model

	△S (cm)	△B_1_ (cm)	△X (cm)	△B_2_ (cm)	α (°)	β (°)
Wound track A	0.5–0.8	0.4–0.5	11.7^(1)^ + 8.2^(2)^	0.3	125	45
Model C	0.1^(3)^ + 0.5^(4)^	0.5	13.5 ^(5)^	0.5	125	50^(6)^

^(1)^ Distance between the bone walls along the trajectory

^(2)^ Path length in soft tissues after the ricochet on the bone wall

^(3)^ 0.1 cm cowhide, semi-finished chrome tanned upholstery “crust”[27]

^(4)^ Gelatine 10%

^(5)^ Distance between the polyurethane walls of the sphere along a straight trajectory

^(6)^ Expected value of the incident angle β on the second polyurethane wall for a straight trajectory

As for the “open shape” model, the thickness of each single layer in the model should correspond approximately to the thickness of the corresponding human tissue measured using the PMCT images considering the wound track position (Fig. 1 and Table 1).

However, the “spherical shape” model represents some limitations; in fact, its spherical form does not allow a whole control on the parameters related to the trajectory. This makes it therefore not obvious to accurately reproduce the desired impact angles and an established penetration length at the same time, affecting heavily the results. Therefore, only the wound track A has been simulated by the mean of the “spherical” model (Table 3). This point will be discussed further in the document.

Two shots have been performed on this model to simulate the wound A, they were called C.1 and C.2.

### Shooting tests set-up

**Fig. 6:**
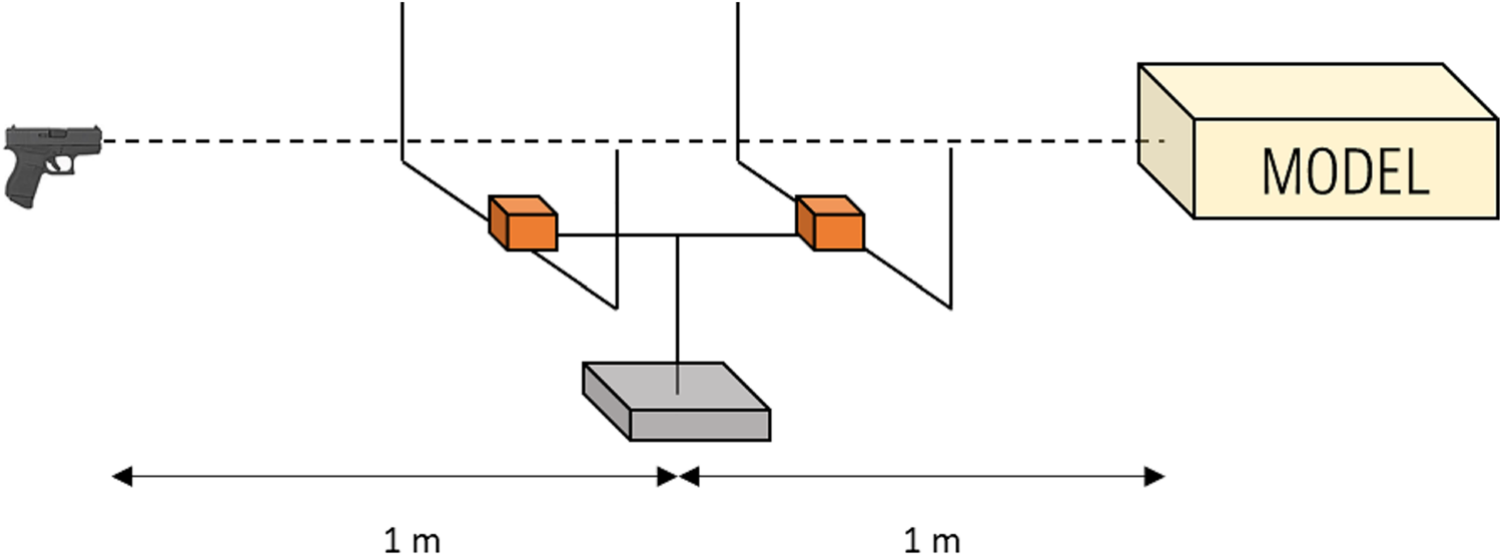
Shooting test set-up

The firearm and the ammunition used for the ballistic tests were all fired with an Astra CUB calibre 6.35 mm Browning (0.25 Auto) semi-automatic pistol with a 5 cm (2″) barrel and a Geco cartridge equipped with a brass Full Metal Jacket (FMJ) projectile weighting 3.2 g (50 grains). The cartridges were of the same brand and type as the one as used in the two cases described above. The pistol was fixed in a ransom rest at 2 m distance from the synthetic model (Fig. 6). A velocity detector (Drello®, Germany) was placed in the middle of the trajectory at 1 m from the pistol and 1 m from the model. For each shot, the velocity has been recorded.

Four shots were performed on the “open shape” models (one shot for each model, namely A.1, A.2, B.1 and B.2) and two shots on the “spherical” model (C.1 and C.2). Before each shot, the temperature of the gelatine within the model was measured with a thermometer at a depth of 4 cm from the upper surface. After the shot, the “open shape” and “spherical” models were first documented photographically and secondly by the mean of a CT-scan. Finally, the projectiles were extracted from the models, photographed and described.

### Comparison between real cases and ballistic tests

**Fig. 7:**
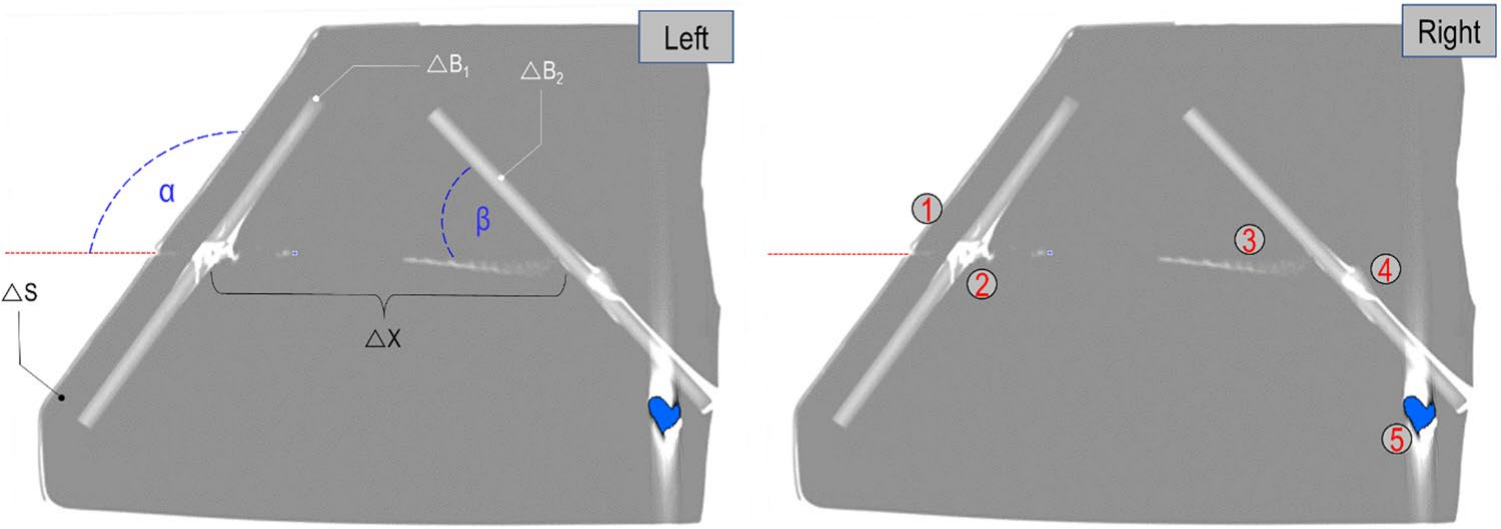
Axial view of the CT-scan images of the Model A.2. Left: representation of the shooting trajectory (red dotted line) as well the shooting parameters α (125°), △S (0.6 cm), △B_1_ (0.5 cm), △X (~ 11 cm), △B_2_ (0.5 cm), and β (43°) (for more details see Figs. 1 and 3). Right: the same CT-image as in A) with additional information: (1) entry hole, (2) hole in the first polyurethane wall with funnel form, (3) track in the gelatine attesting the side position of the projectile, (4) fracture in the second polyurethane wall, (5) position of the projectile after ricochet (in blue)

The findings observed visually on the models were completed with the observations and measurements done by using the CT-scan after the shooting. An example of the measurements and observation by the mean of the CT images is presented in Fig. 7.

The results of the synthetic models were then compared with the observations done during the autopsy of the two victims and especially the observations done by analysing the PMCT. The following characteristics observed on the synthetic models have been noted and compared to the corresponding findings of the wound tracks of victims A and B: damage on the first polyurethane layer (first impact on the skull wall of the victims), length and characteristics of the wound track in the gelatine (in the brain of the victims), results of the interaction between the bullet and the second polyurethane layer (second impact on the inner part of the skull wall of the victims, ricochet), the bullet position and the bullet deformation.

## Results

### Data recorded during the ballistic tests

Before each shot, the temperature of the gelatine has been measured in order to assure that it was laying in an acceptable range (not more than 4°). During the ballistic tests, the velocity of each projectile has been measured (Fig. 6). These values have been noted and summarized in Table 4.

**Table 4:** Velocity of the projectiles before impact against the model and temperature of the gelatine during the ballistic tests

Model	V_1_ (m/s)	T_Gelatine_ (°C)
A.1	222.2	3
A.2	205.7	3
B.1	219.6	3
B.2	216.6	3
C.1	215.1	4
C.2	223.9	4

### Bullet retention and deformation

**Fig. 8:**
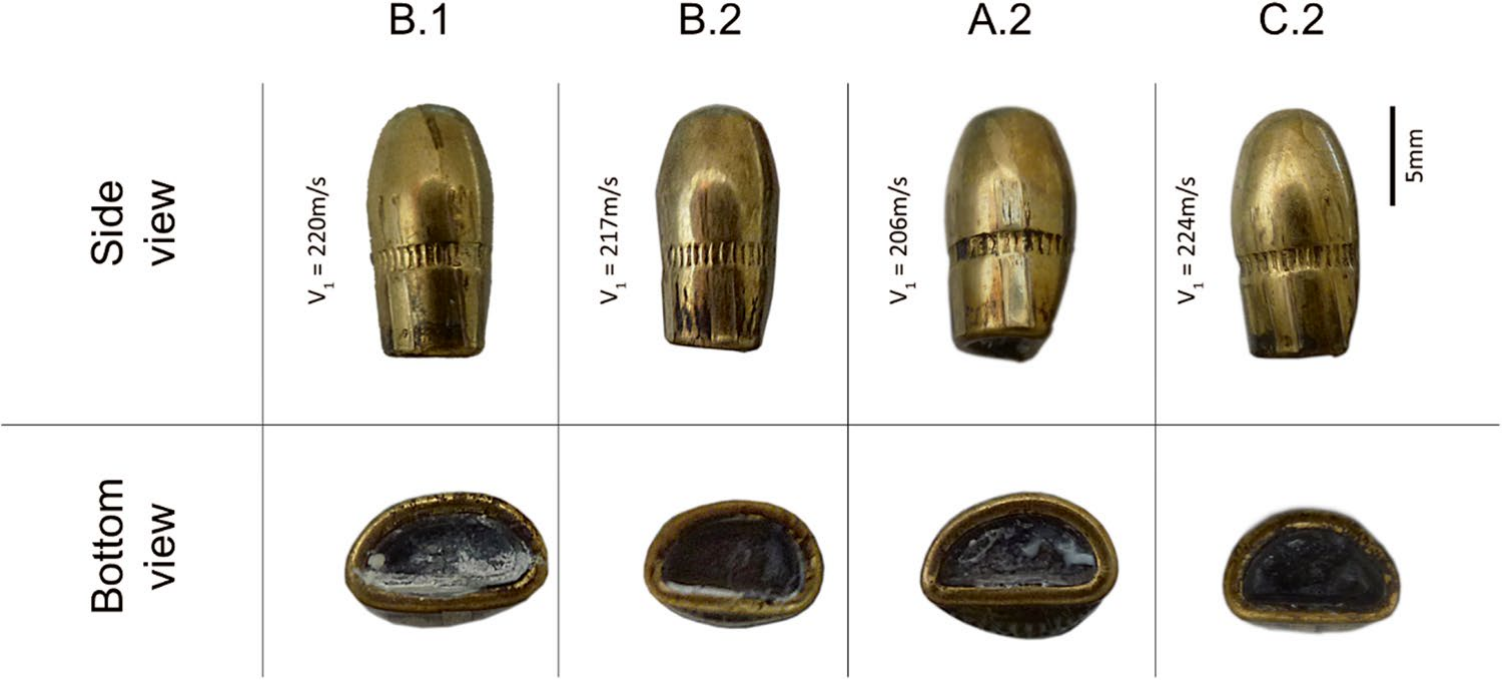
From left to right projectiles recovered respectively from the models B.1, B.2, A.2 and C.2 (side view (first row) and bottom view (second row))

With the exception of the models A.1 and C.1, the projectiles were retained in the models. The ones which were retained in the models were recovered from the models after the photographical documentation and the CT-Scan which was performed. All projectiles showed a main side deformation close to the base (Fig. 8). This observation is in concordance to Riva et al. [1], according to whom the bullets are flattened at the base because they move to the side position before impacting the second polyurethane layer.

### Comparison between real cases and ballistic tests

**Fig. 9:**
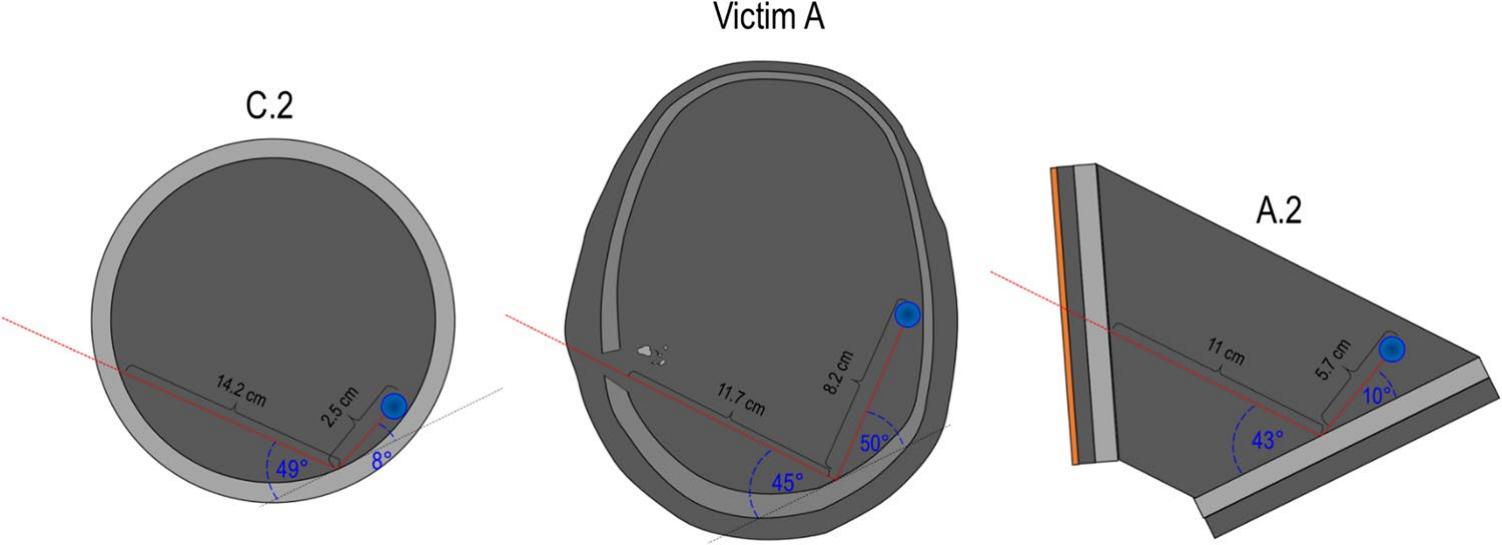
Comparison between the “spherical model” C.2, the wound track of victim A and the “open shape” model A.2. For each drawing, the following information is reported: the wound track length between the two bone walls, the incident angle (β), the ricochet angle and the wound track length after ricochet

**Fig. 10:**
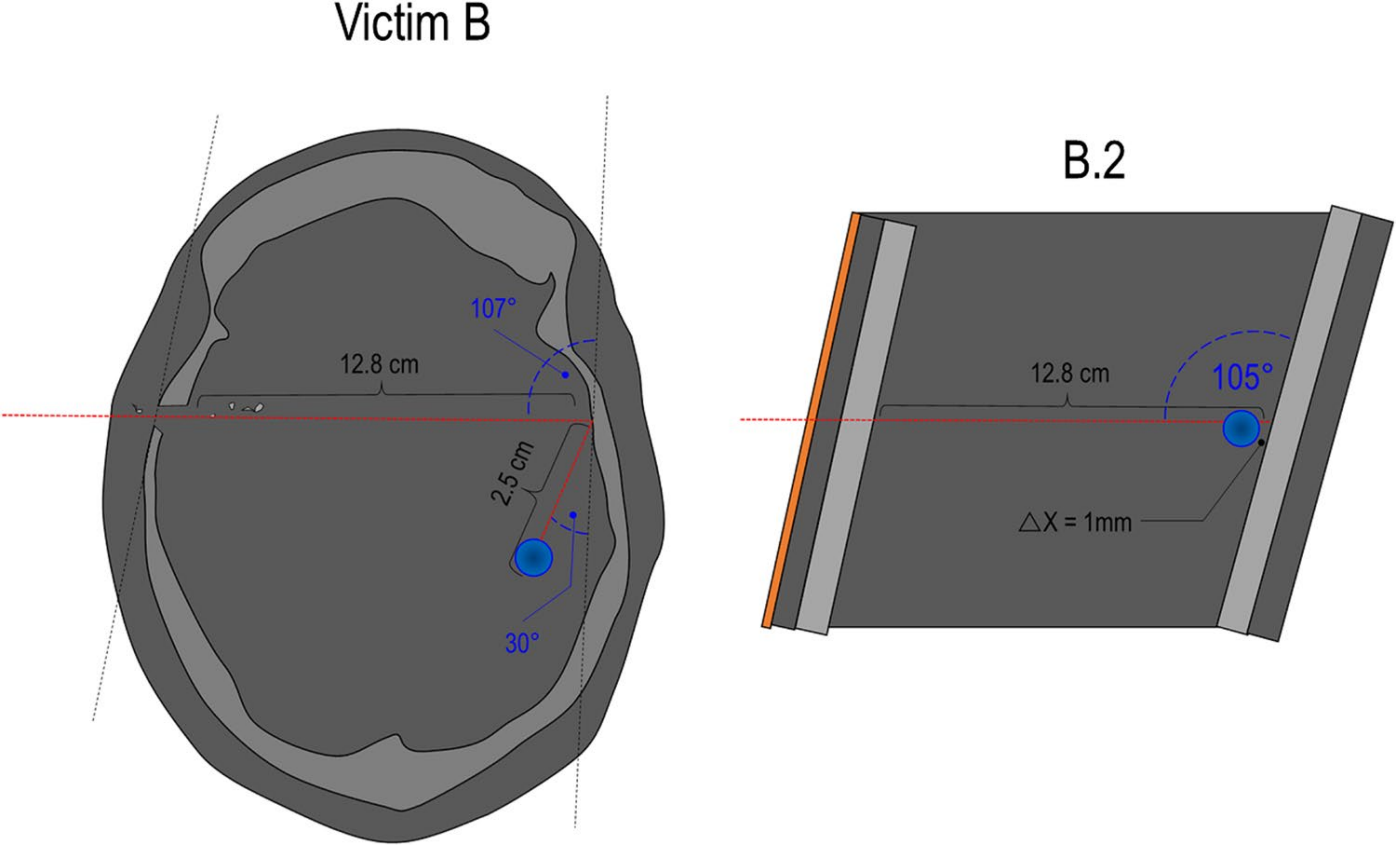
Comparison between the wound track of victim B and the “open shape” model B.2. For each drawing, the following information is reported: the wound track length between the two bone walls, the incident angle (β), the ricochet angle and the wound track length after ricochet

Tables 5 and 6 summarize the most pertinent characteristics observed on the synthetic models as well as the corresponding findings on the wound tracks respectively of victims A and B. The results are represented in Tables 5 and 6; they are completed by a comparison between the models and wound tracks under the form of drawings (Figs. 9 and 10).

**Table 5:** Comparison between findings observed on the models A.1, A.2, C.1, C.2 and the wound track A characteristics

	Wound track length between the two bone walls	Incident angle (β) on the 2° bone wall	Ricochet angle	Wound track length after ricochet	Bullet deformation (Fig. 8)
Model A.1	11.5 cm	47°	Perforation	-	Not recovered
Model A.2	11.0 cm	43°	10°	5.7 cm	Flattened at the base
Wound track A	11.7 cm	~ 45°	~ 50°	8.2 cm	Small deformations on its body and a main deformation at the base (flattened)
Model C.1	15.3 cm	~ 59°	Perforation	-	Not recovered
Model C.2	14.2 cm	~ 49°	8°	2.5 cm	Flattened at the base

**Table 6:** Comparison between findings observed on the models B.1, B.2 and the wound track B characteristics

	Wound track length between the two bone walls	Incident angle (β) on the 2° bone wall	Ricochet angle	Wound track length after ricochet	Bullet deformation (Fig. 8)
Model B.1	12.5 cm	75°	2° polyurethane layer perforated/projectile retained by the model	-	Flattened at the base
Model B.2	12.8 cm	77°	Unknown	0.1 cm	Flattened at the base
Wound track B	12.8 cm	~ 73°	~ 30°	~ 2.5 cm	Small deformations on its body and a main deformation at the base (flattened)

## Discussion

Two interesting shooting cases that took place under similar circumstances were submitted to our legal medicine institute. After shooting, both victims were brought to the hospital, where they died shortly later. For both victims an ante-mortem, a post-mortem CT and an autopsy were performed. In both cases the main findings were: (1) a FMJ projectile of calibre 6.35 mm Browning retained in the head; (2) CT observations showed a deviated trajectory after an internal ricochet on the skull wall; (3) a discrepancy between projectile position in the ante-mortem and the post-mortem CT caused by bullet migration. The particularity of these two cases and their shared shooting conditions constituted a solid basis to contribute to the existing knowledge on the use of synthetic models in wound ballistic via ballistic tests.

### Considerations regarding bullet migration

Despite that it is a phenomenon described in the literature [35–40], bullet migration is rarely documented in forensic caseworks because, to observe such phenomenon, some conditions which are difficulty given in standard forensic cases have to be fulfilled: (a) the projectile has obviously to be retained and (b) if migration occurs, (c) this last has to be detected. Because of the rarity of bullet migration and even if the study does not focus specially on this phenomenon, the findings related to the projectile’s displacements collected during both cases are briefly analysed and discussed below.

- The projectile has obviously to be retained: the frequency of cases where bullets are retained in the head is relatively low. Especially FMJ projectiles in calibre 9 mm Luger and stronger tend to perforate the skull. Retained projectiles are more often observed in cases involving small calibres with limited muzzle energy. The use of such calibres in our country is limited. In fact, analysing the ballistic cases of our institute between 2015 and 2020, showed that only 17.7% of cases involved a retained projectile in the head (14 cases of 79 cases with headshot). Within these 14 cases, 80% of deaths were caused by a shot with a calibre having a muzzle energy less than approx. 250 Joules (6 mm Flobert, 6.35 mm Browning, 7.65 mm Browning, 0.22LR), like in the two cases described in this document.
- Migration must occur: it is to highlight that bullet migration can be facilitated under some conditions. In the reported cases, both victims survived the headshot and were transported to the hospital. During the period in the hospital, the patients were laid on the back; so that the gravity acted on the bullets mainly in the same direction. Additionally, as the victims survived for a certain time, the brain parenchyma along the wound track got weaken and brain swelling took place. Such a weakness could influence the consistence of the wound path and together with the gravity it could favour a bullet displacement. In our opinion, these are the explanations for the bullet migration in our two reported cases.
- The migration has to be detected: as the two reported cases were transported to the hospital and a CT-scan (ante-mortem) was performed on both of them at their arrival to the hospital, we have the chance to have the original position of the bullet documented. As both victims were transported to our institute after death, a post-mortem CT-scan was performed two to three days after the shooting. Having the images of both CT-scans available is quite rare. In our two cases, it permitted to compare the bullet position in detail.

### Simulants characteristics

The models developed and used in this study (“open shape” and “spherical”) have been inspired respectively by Riva et al. [1] and Thali et al. [33]; both have been composed using only materials, which are well known in wound ballistics tests; namely a layer of leather [27], the ballistic gelatine 10% at 4° [22, 28, 32, 44–46] and polyurethane plates [1, 5, 14, 19].

Various studies can be found in the wound ballistics literature regarding the quantitative and qualitative aspects of *skin* perforation [27, 47–49]. However, few studies propose concretely a suitable material, which can be used to simulate the skin; the difficulty to find a simulant which fulfils simultaneously the quantitative as well as the qualitative needs can be one of the reasons. Synthetically and natural materials have been tested by Jussilla et al. [27]. According to the results obtained by the authors, the best natural simulant was the “semi-finished chrome tanned upholstery “crust” cowhide”. This skin simulant has already been used successfully in wound ballistics research [9].

When headshots are analysed, questions arise about the most suitable soft tissue surrogate to simulate the *brain parenchyma*. Soft tissues surrogates have already been widely investigated; the most accepted one in the forensic field is the ballistic gelatine at 10% of concentration used at 4 °C temperature (FBI standard) [22, 32]. Ballistic gelatine at 20% used at 10 °C (NATO formulation) is also widely used in the ballistic domain [22]. Other similar products, like Clearballistics®, a synthetic gelatine, exist and are also commercially available. However, this alternatives does not meet the penetration standards applicable for the ballistic gelatine [50]. Other more specific solutions to simulate brain tissues have also been studied [51]; however, the most widely used in the field of wound ballistics remains the gelatine 10% at 4 °C [1, 5, 33, 52]. Although the ballistic gelatine 10% showed already good reproducibility in several studies [1, 18, 23, 33, 34], it is known that it does not reproduce exactly the characteristics of brain parenchyma and its reaction to the shooting process [53].

Furthermore, different types of polymers have already been tested to simulate *bone* structures. Such type of material has the advantage to successfully reproduce the macroscopic fracture pattern of real bone under a ballistic impact [18, 23, 33, 34]; however, it lacks the complex structure of a real human bone [25]. The most used synthetic proxy in wound ballistics studies seems to be the polyurethane products provided by the Swiss company Synbone® in the form of plates, spheres or cylinders [1, 18–20, 41, 52, 54, 55]. Even if this polymer seems to be a suitable solution to simulate the loss of energy associated to the perforation of a bone structure [14], it does not provide sufficient resistance to reproduce a bullet deformation [1]. According to Kraniotti et al., the polyurethane spheres of Synbone® can be used to simulate the biggest portion of the cranial vault, but are not appropriate for the facial skeleton, which exhibits considerable anatomical complexity [52].

The obtained results during this study showed a good potential of the different compounds when assembled into a unique model. The comparison between the findings on victim A and the results in the Model A.2 showed in terms of penetration and bullet behaviour a good correlation. However, even if the gelatine and the leather have already been tested and validated as simulants, a real suitable bone simulant does not yet exist. In fact, the results showed a limited projectile deformation compared to the questioned ones (Fig. 2 vs. Figure 8). A similar result was already observed by Riva et al. [1]. The suboptimal bone simulant can lead to differences between the wound track characteristics and the bullet deformation observed in real cases and in ballistic tests. The projectile deformation caused by the bone’s interaction should be one priority taken into account in developing new bone simulants.

However, the selected compounds have the great advantage to be homogeneous allowing to reach a good level of reproducibility. This allows to keep the set-up unchanged and to modify only single parameters permitting to analyse them further in detail. On the other hand, the homogeneity of the synthetic materials can also be a disadvantage. Human tissues are not as homogenous and can therefore react differently. The difference in the homogeneity of the selected compounds used and the human tissues can explain some differences which we have noted between our expected and our finally obtained results. For example, the surface irregularities on the inner part of the skull wall can more easily, in case of ricochet, generate different angles of rebound than the smooth surface of the polyurethane plates or spheres. This might explain the discrepancy found between the wound track and its extent of injury of victim A and the experimental results concerning the simulated wound track of it. In victim A, the angle of ricochet was about 50° (Table 5) compared to the limited ricochet angle observed after the shot performed on the model A.2 (ricochet angle about 10°). Such difference can also be enhanced by the limited bullet deformation caused by the interaction with the first polyurethane layer.

Finally, it should also be noted that some differences between the real wound tracks and the models simulations could be related to the ammunition batch and the questioned pistol, as also the exact shooting distance, who’s exact information were not available at the moment of the ballistic tests. In the opinion of the authors despite these uncertainties, their influence on the results remains limited from a wound ballistics point of view.

### Comparison of the “open shape” and the “spherical” model

Regarding the comparison between the two types of models, the “open shape” model allowed to better control all the shooting parameters. This aspect was not always fulfilled when using the “spherical” model. Even a small error from the intended point of impact on a 19 cm sphere leads to an impact angle that is bigger or smaller than intended. On the other side, the “open shape” model used in this study has been tested only with low energy projectiles [1]. With such ammunition, the energy deposition and the temporary cavity are limited, especially when FMJ projectiles are used. The effect of a bigger temporary cavity has not been tested but it is possible to imagine that a “spherical” model should be more suitable to simulate the damages caused by more powerful projectiles into the head, as the spheres enclose the gelatine better than an open model does. This point should be further investigated if simulations with such ammunition are taking into account. Moreover, the polyurethane walls of the “spherical” model are provided with constant thickness (available in 5, 6 and 7 mm); these values can be hardly modified. It is thus difficult to deal with cases where the thickness of the bone wall changes considerably for the same victim, depending on its locations, like it’s the case for the skull. It is important to keep in mind that the “open” shape model allows a clear visualisation of the bullet behaviour and the tissues reaction during the shooting if a High-Speed camera is used.

### Criticism around the bone simulant and further research

Wound tracks, from Victim A and B, and their injury effect have been successfully reproduced by the mean of the “open shape” models and the wound track of victim A has been also reproduced similarly by using the “spherical” model. Concerning all the retained shots, the main difference was the bullet deformation, which was less pronounced in our ballistic tests compared to the projectiles found in the head of the victims. This difference is probably caused by the physical properties of the Synbone® polyurethane plates which might under-represent the strength and/or the density of human bone. Despite a simulant does not need to have exactly the same biomechanical properties as the human tissue it represents, it has to provide results, which can be extrapolated or scaled in a constant way [28, 56]. However, this aspect cannot be applied by using Synbone® polyurethane plates when considering the deformation of the projectile. In fact, the path of a deformed projectile in soft tissues will not be the same in terms of damages and penetration’s length as a path left by an “intact” projectile. The magnitude of the bullet’s deformation can have important consequences on the wounding profile and wound track [1]. This aspect should thus not be underestimated and should be strongly considered for the development of more adapted materials.

## Conclusion

In order to reconstruct shooting incidents, ballistics tests based on simulants are often recommended in order to reproduce the wound characteristics of the victims. The validation of such simulants needs still to be improved through the comparison with real caseworks. In this study, the findings observed during various investigations (ante- and post-mortem CT-Scan, autopsy) performed on two victims killed in the same shooting have been reproduced with shooting tests on synthetic models.

Two different models have been assembled to simulate the victim’s head (“open shape” and “spherical” model). Both are composed by a layer of leather, two Synbone® polyurethane layers and ballistic gelatine 10% to simulate respectively the skin, the bone walls of the skull and the brain parenchyma. In the first model, named “open shape” model, the three compounds have been assembled sequentially layer by layer without lateral walls to enclose them; as opposed to the second model, namely the “spherical” model, which was enclosed by a polyurethane sphere.

The comparison between the investigation findings of the victims and the shooting tests results of both models shows a good correlation in terms of projectile penetration and bullet behaviour. However, the “open shape” model allowed to better control all the shooting parameters, though it has been tested until now only with low energy projectiles.

The main difference between the real cases and our models was the bullet deformation, which was less pronounced in the ballistic tests compared to the projectiles found in the head of the victims. This result could be related to the bone simulant, which we used, which might under-represent the strength and density of the human bone. Future studies which target the development and the validation of ballistic bone simulants should thus focus on this aspect in priority.

## Data and materials availability

Under request, all anonymous data can be asked to the corresponding author.

## Data Availability

Not applicable.

## References

[1] Riva F, Lombardo P, Zech WD, Jackowski C, Schyma C (2019). Individual synthetic head models in wound ballistics — a feasibility study based on real cases. Forensic Sci Int.

[2] Colard T, Delannoy Y, Bresson F, Marechal C, Raul JS, Hedouin V (2013). 3D-MSCT imaging of bullet trajectory in 3D crime scene reconstruction: two case reports. Leg Med (Tokyo).

[3] Bresson F, Franck O (2010). Comparing ballistic wounds with experiments on body simulator. Forensic Sci Int.

[4] Maiden N (2009). Historical overview of wound ballistics research. Forensic Sci Med Pathol.

[5] Kneubuehl BP, Coupland RM, Rotschild MA, Thali MJ (2011). Wound Ballistics - Basics and Applications.

[6] Fackler ML (1988). Wound ballistics: a review of common misconceptions. JAMA.

[7] Maio VJMd (1999) Gunshot Wounds — Practical Aspects of Firearms, Ballistics, and Forensic Techniques, Seond Edition ed., CRC Press, Boca Raton, New York

[8] Riva F, Kerkhoff W, Bolck A, Mattijssen E (2017). Possible influences on bullet trajectory deflection in ballistic gelatine. Forensic Sci Int.

[9] Kerkhoff W, Bolck A, Alberink I, Mattijssen E, Hermsen R, Riva F (2018). Pistol bullet deflection through soft tissue simulants. Forensic Sci Int.

[10] Kerkhoff W, Mattijssen E, Riva F (2020). Influence of bullet type and muzzle-to-target distance on trajectory deflection through a soft tissue simulant. Forensic Sci Int.

[11] Riva F, Mattijssen E, Kerkhoff W (2018). Rifle bullet deflection through a soft tissue simulant. Forensic Sci Int.

[12] Wightman G, Beard J, Allison R (2010). An investigation into the behaviour of air rifle pellets in ballistic gel and their interaction with bone. Forensic Sci Int.

[13] Pollak S, Rothschild MA (2004). Gunshot injuries as a topic of medicolegal research in the German-speaking countries from the beginning of the 20th century up to the present time. Forensic Sci Int.

[14] Henwood BJ, Appleby-Thomas G (2019). The suitability of Synbone® as a tissue analogue in ballistic impacts. J Mater Sci.

[15] Bolliger SA, Poschmann SA, Thali MJ, Eggert S (2017). A fully synthetic lung model for wound-ballistic experiments—first results. Forensic Sci Int.

[16] Humphrey C, Kumaratilake J (2016). Ballistics and anatomical modelling — a review. Leg Med (Tokyo).

[17] Breeze J, Carr DJ, Mabbott A, Beckett S, Clasper JC (2015). Refrigeration and freezing of porcine tissue does not affect the retardation of fragment simulating projectiles. J Forensic Leg Med.

[18] Mahoney PF, Carr DJ, Miller D, Teagle M (2017). The effect of helmet materials and simulated bone and tissue layers on bullet behaviour in a gelatine model of overmatch penetrating head injury. Int J Legal Med.

[19] Bir C, Andrecovich C, DeMaio M, Dougherty PJ (2016). Evaluation of bone surrogates for indirect and direct ballistic fractures. Forensic Sci Int.

[20] Smith MJ, James S, Pover T, Ball N, Barnetson V, Foster B, Guy C, Rickman J, Walton V (2015). Fantastic plastic? Experimental evaluation of polyurethane bone substitutes as proxies for human bone in trauma simulations. Leg Med (Tokyo).

[21] Schyma C, Madea B (2012). Evaluation of the temporary cavity in ordnance gelatine. Forensic Sci Int.

[22] Maiden NR, Fisk W, Wachsberger C, Byard RW (2015). Ballistics ordnance gelatine - How different concentrations, temperatures and curing times affect calibration results. J Forensic Leg Med.

[23] Mahoney PF, Carr DJ, Delaney RJ, Hunt N, Harrison S, Breeze J, Gibb I (2017). Does preliminary optimisation of an anatomically correct skull-brain model using simple simulants produce clinically realistic ballistic injury fracture patterns?. Int J Legal Med.

[24] Mahoney P, Carr D, Harrison K, McGuire R, Hepper A, Flynn D, Delaney RJ, Gibb I (2019). Forensic reconstruction of two military combat related shooting incidents using an anatomically correct synthetic skull with a surrogate skin/soft tissue layer. Int J Legal Med.

[25] Mahoney P, Carr D, Arm R, Gibb I, Hunt N, Delaney RJ (2018). Ballistic impacts on an anatomically correct synthetic skull with a surrogate skin/soft tissue layer. Int J Legal Med.

[26] Mabbott A, Carr DJ, Champion S, Malbon C (2016). Comparison of porcine thorax to gelatine blocks for wound ballistics studies. Int J Legal Med.

[27] Jussila J, Leppaniemi A, Paronen M, Kulomaki E (2005). Ballistic skin simulant. Forensic Sci Int.

[28] Jussila J (2004). Preparing ballistic gelatine—review and proposal for a standard method. Forensic Sci Int.

[29] Jin Y, Mai R, Wu C, Han R, Li B (2018). Comparison of ballistic impact effects between biological tissue and gelatin. J Mech Behav Biomed Mater.

[30] Grosse Perdekamp M, Pollak S, Thierauf A, Strassburger E, Hunzinger M, Vennemann B (2009). Experimental simulation of reentry shots using a skin-gelatine composite model. Int J Legal Med.

[31] Carr DJ, Stevenson T, Mahoney PF (2018). The use of gelatine in wound ballistics research. Int J Legal Med.

[32] Fackler ML, Malinowski JA (1988). Ordnance gelatine for ballistic studies. Am J Forens Med Pathol.

[33] Thali MJ, Kneubuehl BP, Zollinger U, Dirnhofer R (2002). The ‘‘Skin–skull–brain model’’: a new instrument for the study of gunshot effects. Forensic Sci Int.

[34] Carr D, Lindstrom AC, Jareborg A, Champion S, Waddell N, Miller D, Teagle M, Horsfall I, Kieser J (2015). Development of a skull/brain model for military wound ballistics studies. Int J Legal Med.

[35] Rapp LG, Arce CA, McKenzie R, Darmody WR, Guyot DR, Michael DB (2016). Incidence of intracranial bullet fragment migration. Neurol Res.

[36] Rammo RA, De Fazio MV, Bullock MR (2012). Management of migrating intracranial bullets: lessons learned from surviving an AK-47 bullet through the lateral brainstem. World Neurosurg.

[37] Chute DJ, Newman K, Bready RJ, Benjamin ED (2017). Case report of a migrating bullet: an unusual cause of postmortem confusion. J Forensic Sci.

[38] Chan YT, Al-Mahfoudh R, Thennakon S, Eldridge P, Pillay R (2015). Migrating intrathecal high-velocity projectile. Br J Neurosurg.

[39] Bachinger D, Bolliger S, Huber GF, Laske RD (2015). Ballistic reconstruction of a migrating bullet in the parapharyngeal space. Case Rep Otolaryngol.

[40] Avci SB, Acikgoz B, Gundogdu S (1995). Delayed neurological symptoms from the spontaneous migration of a bullet in the lumbosacral spinal canal. Case report, Paraplegia.

[41] Pullen A, Kieser DC, Hooper G (2020) A study into the viability of Synbone(R) as a proxy for Sus scrofa (domesticus) ribs for use with 5.56-mm open tip match ammunition in ballistic testing, Int J Legal Med 10.1007/s00414-020-02416-8 32864715

[42] Thali MJ, Kneubuehl BP, Dirnhofer R (2002). A "skin-skull-brain model" for the biomechanical reconstruction of blunt forces to the human head. Forensic Sci Int.

[43] Thali MJ, Kneubuehl BP, Zollinger U, Dirnhofer R (2003). A high-speed study of the dynamic bullet–body interactions produced by grazing gunshots with full metal jacketed and lead projectiles. Forensic Sci Int.

[44] Mattijssen EJ, Alberink I, Jacobs B, van den Boogaard Y (2016). Preservation and storage of prepared ballistic gelatine. Forensic Sci Int.

[45] Maiden NR, Musgrave I, Fisk W, Byard RW (2016). Pig organ energy loss comparison experiments using BBs. J Forensic Sci.

[46] Jussila J (2005). Measurement of kinetic energy dissipation with gelatine fissure formation with special reference to gelatine validation. Forensic Sci Int.

[47] Bir CA, Resslar M, Stewart S (2012). Skin penetration surrogate for the evaluation of less lethal kinetic energy munitions. Forensic Sci Int.

[48] Mattoo BN (1984). Discussion of "Minimal velocities necessary for perforation of skin by air gun pellets and bullets". J Forensic Sci.

[49] Tausch D, Sattler W, Wehrfritz K, Wehrfritz G, Wagner HJ (1978). Experiments on the penetration power of various bullets into skin and muscle tissue. Z Rechtsmed.

[50] Courtney E, Courtney A, Andrusiv L, Courtney M (2017) Clear Ballistics Gel®: High Speed Retarding Force Analysis of Paraffin-Based Alternative to Gelatin-based Testing of Lead-Free Pistol Bullets

[51] Falland-Cheung L, Waddell JN, Lazarjan MS, Jermy MC, Winter T, Tong D, Brunton PA (2017). Use of agar/glycerol and agar/glycerol/water as a translucent brain simulant for ballistic testing. J Mech Behav Biomed Mater.

[52] Taylor SC, Kranioti EF (2018). Cranial trauma in handgun executions: experimental data using polyurethane proxies. Forensic Sci Int.

[53] Lazarjan M, Geoghegan P, Jermy M, Taylor M (2014). Experimental investigation of the mechanical properties of brain simulants used for cranial gunshot simulation. Forensic Sci Int.

[54] Sterzik V, Kneubuehl BP, Bohnert M, Riva F, Glardon M (2017). Bullet fragmentation preceding a contour shot: case study and experimental simulation. Int J Legal Med.

[55] Thali MJ, Kneubuehl BP, Zollinger U, Dirnhofer R (2002). The "skin-skull-brain model": a new instrument for the study of gunshot effects. Forensic Sci Int.

[56] Janzon B, Schantz B, Seeman T (1988). Scale Effects in Ballistic Wounding.

